# Supplementary figures and images for: Monosomy 18p with Unbalanced Translocation Between 13 and 18 Chromosomes: First Reported Case in Serbia
Source: Diagnostics (Basel). 2025 Feb 4;15(3):358. doi: 10.3390/diagnostics15030358 (PMC11817255; doi:10.3390/diagnostics15030358)

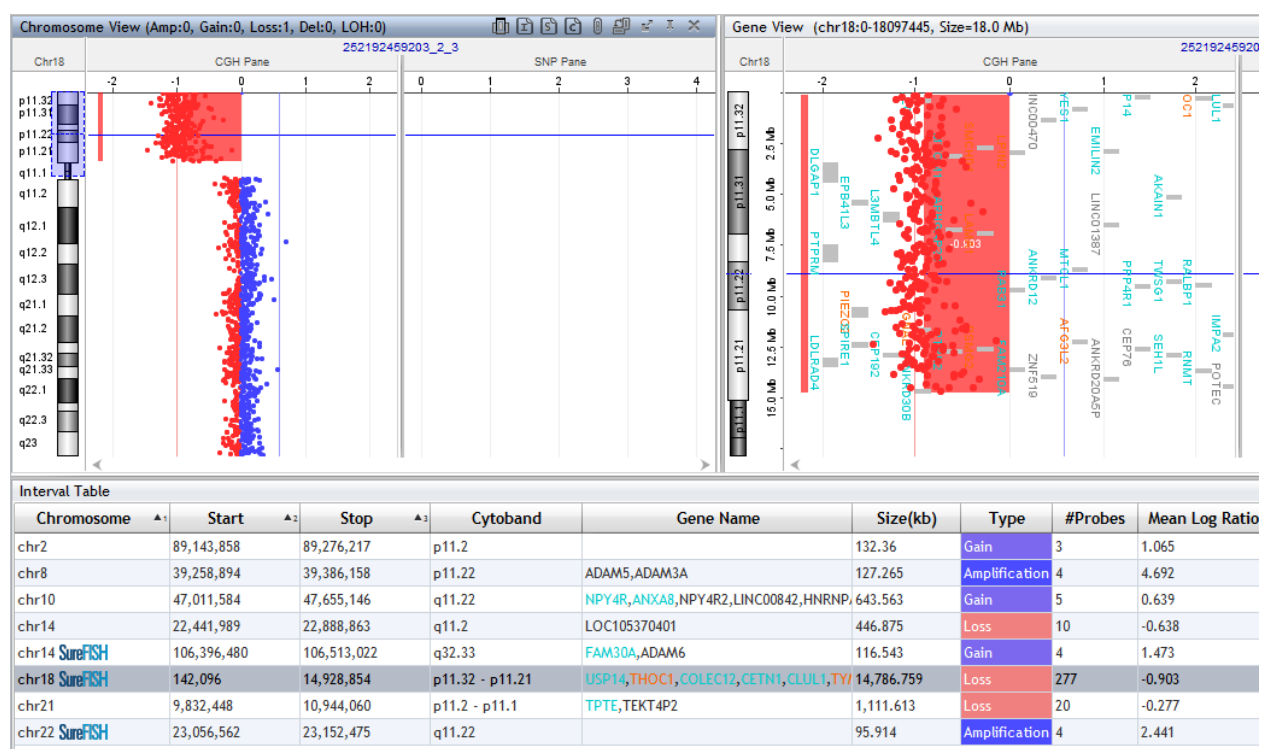

Figure S1. Array-CGH profiles analysis using Agilent CytoGenomic Analytics software.

Supplement: Supplementary file 1 [file diagnostics-15-00358-s001.zip › diagnostics-3358782-supplementary.pdf]
